# Motion-Guided Deep Image Prior for Cardiac MRI

**Published:** 2024-12-05

**Authors:** Marc Vornehm, Chong Chen, Muhammad Ahmad Sultan, Syed Murtaza Arshad, Yuchi Han, Florian Knoll, Rizwan Ahmad

**Affiliations:** * Artificial Intelligence in Biomedical Engineering, Friedrich-Alexander-Universität Erlangen-Nürnberg, Erlangen, Germany; † Biomedical Engineering, The Ohio State University, Columbus, OH, USA; ‡ Magnetic Resonance, Siemens Healthineers AG, Erlangen, Germany; § Division of Cardiovascular Medicine, The Ohio State University, Columbus, OH, USA

## Abstract

Cardiovascular magnetic resonance imaging is a powerful diagnostic tool for assessing cardiac structure and function. Traditional breath-held imaging protocols, however, pose challenges for patients with arrhythmias or limited breath-holding capacity. We introduce Motion-Guided Deep Image prior (M-DIP), a novel unsupervised reconstruction framework for accelerated real-time cardiac MRI. M-DIP employs a spatial dictionary to synthesize a time-dependent template image, which is further refined using time-dependent deformation fields that model cardiac and respiratory motion. Unlike prior DIP-based methods, M-DIP simultaneously captures physiological motion and frame-to-frame content variations, making it applicable to a wide range of dynamic applications. We validate M-DIP using simulated MRXCAT cine phantom data as well as free-breathing real-time cine and single-shot late gadolinium enhancement data from clinical patients. Comparative analyses against state-of-the-art supervised and unsupervised approaches demonstrate M-DIP’s performance and versatility. M-DIP achieved better image quality metrics on phantom data, as well as higher reader scores for in-vivo patient data.

## 1 Introduction

Cardiovascular magnetic resonance imaging (CMR) is a well-established modality that provides comprehensive structural and functional assessment of the heart. Data acquisition in CMR is typically synchronized with an electrocardiogram (ECG) signal and performed during breath-holds. However, this strategy is not feasible for patients who cannot hold their breath or have arrhythmias. Real-time free-breathing imaging offers an alternative but requires high acceleration rates, leading to compromised image quality with reduced spatial and temporal resolution. Improving the quality of real-time imaging has been an active area of research [1].

Sparsity-based compressed sensing (CS) has shown great promise for accelerating various MRI applications [2]. More recently, deep learning (DL)-based methods have surpassed CS in image quality [3]–[5]. In particular, end-to-end variational network (VarNet) and similar methods have emerged as a quality benchmark for MRI reconstruction [6]–[8]. However, these methods require large fully sampled datasets for training, which are unavailable for most cardiac applications. Even when available [9], these training datasets are collected under breath-holds, from subjects with regular heart rhythm, and within a narrow range of imaging parameters, including resolution, contrast, and sampling pattern. This limits their adaptability to free-breathing imaging, for imaging arrhythmic patients, and to situations where imaging parameters (e.g., resolution) are vastly different from the ones used in the training dataset.

Unsupervised and self-supervised learning strategies have shown great promise for applications where uncorrupted training data are unavailable. These methods reconstruct images by leveraging redundancies in the undersampled data. Traditional GRAPPA [10] and its nonlinear extension RAKI [11] exemplify self-supervised learning approaches, where relationships between neighboring k-space samples are first learned from the fully sampled autocalibration signal region and then applied to the undersampled regions of k-space. Recently, Yaman et al. introduced a calibration-free method, called Self-Supervised learning via Data Undersampling (SSDU) [12], which divides undersampled k-space data into two subsets. A reconstruction network is trained by taking an aliased image from one subset as input and generating a reconstructed image consistent with the other subset. Once trained on a collection of undersampled measurements, this network can reconstruct images from unseen undersampled data. Although this approach can be naively extended to dynamic applications, it does not exploit the structure in the temporal dimension.

Deep Image Prior (DIP) offers another unsupervised learning framework for solving a wide range of inverse problems [13]. DIP is instance-specific, i.e., the training is performed from scratch for each set of measurements. In DIP, a generative network is trained to map a random code vector to an output consistent with the measurements. A key feature of DIP is that the network structure acts as an implicit prior, obviating the need to use an explicit regularization term. A straightforward extension of DIP for dynamic problems involves training a single network to take a different random code vector at each time step and map it to an image frame consistent with the measurements. While this approach leverages redundancy across frames by employing a common network, it does not fully capture the structure along the temporal dimension.

More recently, extensions of DIP that specifically leverage the temporal structure for dynamic MRI have been proposed. Yoo et al. [14] proposed Time-Dependent Deep Image Prior (TD-DIP), in which the code vectors are an ordered sequence of points on a specifically designed manifold. This approach, however, requires an a priori estimation of the cardiac phase, which can be inferred from a proprietary ECG signal or directly from k-space in the case of a radial or spiral acquisition scheme. In a related line of work, Zou et al. proposed Gen-SToRM [15] for spiral real-time cine imaging. Instead of hand-crafting the manifold in the latent domain, this approach discovers the manifold by directly optimizing the latent code vectors along with the network parameters. Further, temporal regularization is added to the code vectors to enforce smoothness on the manifold. After convergence, the latent code vectors represent the manifold for cardiac and respiratory motion. The follow-up 3D approach MoCo-SToRM [16] models the image series as a single static 3D template image that is warped by time-dependent deformation fields. Instead of image frames, the generator network outputs the deformation fields, and the voxels of the template image are considered trainable parameters and optimized along with the network parameters and code vectors. This approach was designed for 3D radial lung MRI with a focus on respiratory motion only. More recent DIP approaches for cardiac cine MRI reconstruction by Ahmed et al., called DEBLUR [17], and Hamilton et al., called LR-DIP [18], model the cardiac cine series as a low-rank system, where two separate neural networks are used to generate the 2D spatial and the 1D temporal basis, respectively.

In this work, we present Motion-Guided Deep Image Prior (M-DIP). Unlike MoCo-SToRM, M-DIP generates a spatial dictionary comprising multiple elements and synthesizes a time-dependent template image through a weighted combination of the spatial dictionary elements. Then, M-DIP models physiological motion as time-dependent deformation fields applied to the template image. The capability of M-DIP to model both motion and frame-to-frame content changes allows it to be applicable to a wide range of dynamic applications. We validate and evaluate M-DIP using data from the MRXCAT cine phantom [19], free-breathing real-time cine, and single-shot late gadolinium enhancement (LGE).

## 2 Methods

### 2.1 DIP for dynamic applications

For an arbitrary variable (⋅), we denote its temporal sequence as ${\left(\cdot \right)}^{\left(1:T\right)}:={\left\{{\left(\cdot \right)}^{\left(t\right)}\right\}}_{t=1}^{T}$, where T is the total number of frames and t represents the frame index. Using this notation, we represent a sequence of image frames, k-space data, and forward operators, as $x^{\left(1:T\right)}$, $y^{\left(1:T\right)}$, and $A^{\left(1:T\right)}$, respectively, such that

(1) $$y^{\left(t\right)}=A^{\left(t\right)}x^{\left(t\right)}+\epsilon^{\left(t\right)},$$

where $x^{\left(t\right)}\in {\mathbb{C}}^{N}$, $y^{\left(t\right)}\in {\mathbb{C}}^{M}$, $A^{\left(t\right)}\in {\mathbb{C}}^{M\times N}$, and $\epsilon^{\left(t\right)}\in {\mathbb{C}}^{M}$ represent the N-voxel image, multi-coil k-space data, forward operator, and measurement noise, respectively, for the $t^{\text{th}}$ frame.

A straightforward application of DIP for the inverse problem in Equation (1) is to solve the following optimization problem:

(2) $$\hat{\text{Ξ}},{\hat{z}}^{\left(1:T\right)}=\underset{\text{Ξ},z^{\left(1:T\right)}}{\text{arg}\;\text{min}}\sum_{t=1}^{T}{\|A^{\left(t\right)}x^{\left(t\right)}-y^{\left(t\right)}\|}_{2}^{2},\;x^{\left(t\right)}={𝓖}_{\text{Ξ}}\left(z^{\left(t\right)}\right)$$

where ${𝓖}_{\text{Ξ}}:{\mathbb{R}}^{K}\to {\mathbb{C}}^{N}$ is a neural network parameterized by Ξ, and $z^{\left(1:T\right)}$ represents time-dependent code vectors, with $z^{\left(t\right)}\in {\mathbb{R}}^{K}$ for K>0. After training, an arbitrary $t^{\text{th}}$ frame can be recovered by ${\hat{x}}^{\left(t\right)}={𝓖}_{\hat{\text{Ξ}}}\left({\hat{z}}^{\left(t\right)}\right)$. This naive approach, however, does not explicitly model the temporal structure in a dynamic image series $x^{\left(1:T\right)}$.

### 2.2 M-DIP framework

**Figure 1:**
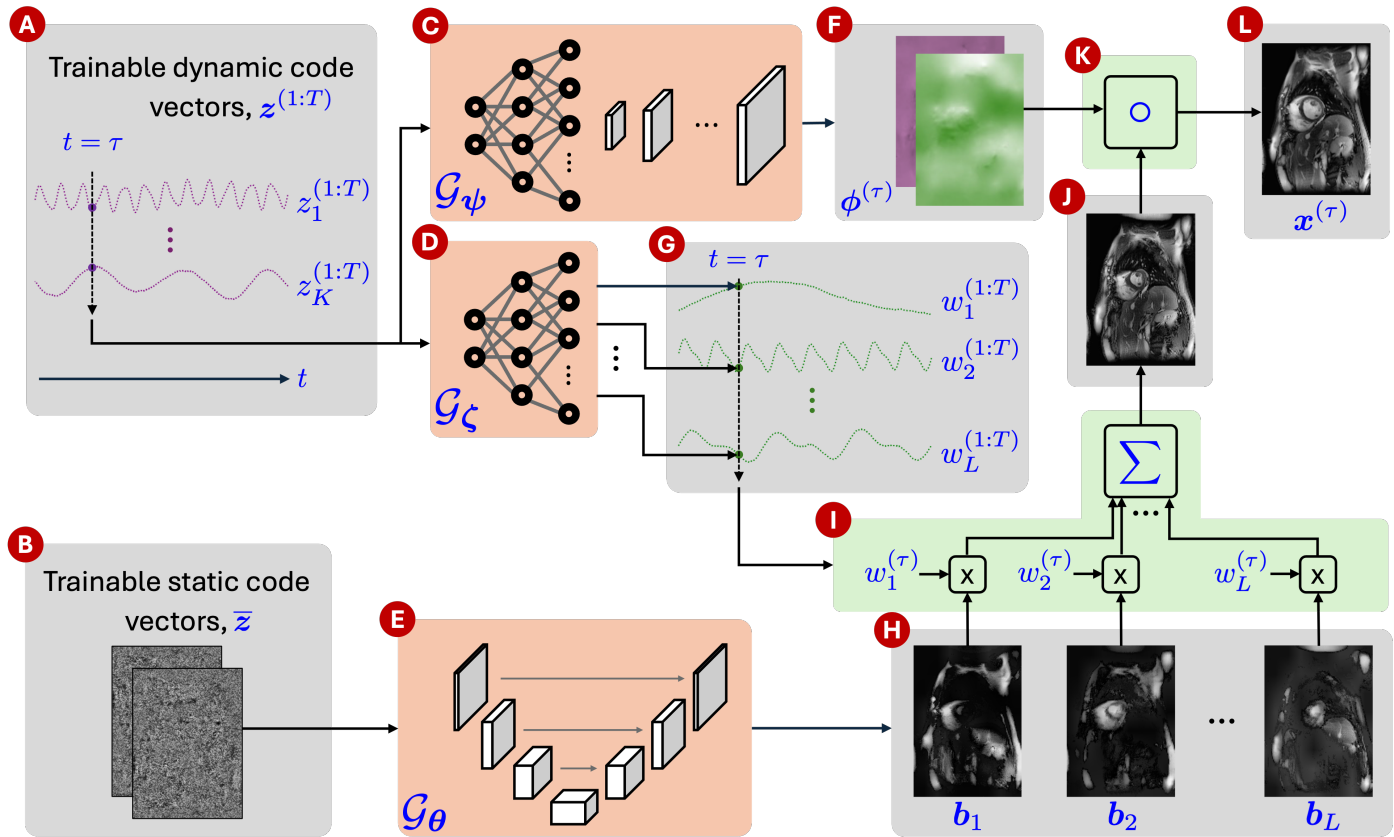
Overview of M-DIP. (A) Trainable dynamic code vectors of dimensionality K, (B) trainable static code vectors of dimensionality c×N, (C) the network ${𝓖}_{\psi }$ that generates a different deformation field for each t, (D) the network ${𝓖}_{\zeta }$ that generates a different set of weights for each t, (E) the network ${𝓖}_{\theta }$ that generates time-invariant L spatial dictionary elements, (F) deformation field generated by ${𝓖}_{\psi }$ at t=τ, (G) weights generated by ${𝓖}_{\zeta }$ at t=τ, (H) spatial dictionary $b_{1:L}$ generated by ${𝓖}_{\theta }$, (I) sum of the dictionary elements after they have been multiplied with time-specific weights, (J) composite template image at t=τ, (K) warping operation that applies deformation to the composite template image, and (L) the output frame at t=τ.

Our proposed method draws inspiration from several previously described DIP approaches for dynamic MRI reconstruction. An overview of the framework is illustrated in Figure 1. In summary, we model a 2D dynamic MRI series as a set of T image frames $x^{\left(1:T\right)}$ such that $x^{\left(\tau \right)}$ at time τ is constructed by warping a composite template image with deformation fields $\phi^{\left(\tau \right)}\in {\mathbb{R}}^{2\times N}$. These time-dependent deformation fields model in-plane cardiac and respiratory motion. However, these fields alone cannot model through-plane motion or contrast changes over time. To model such variations, we synthesize the composite template image from L spatial dictionary elements $b_{1:L}:={\left\{b_{i}\right\}}_{i=1}^{L}$ using weights $w^{\left(1:T\right)}$, where $b_{i}\in {\mathbb{C}}^{N}$ is the $i^{\text{th}}$ element of the spatial dictionary $b_{1:L}$ and $w^{\left(t\right)}\in {\mathbb{C}}^{L}$. Then, each frame $x^{\left(\tau \right)}$ is expressed as

(3) $$x^{\left(\tau \right)}=\phi^{\left(\tau \right)}\circ \underset{\text{composite}\;\text{template}}{\underset{︸}{\sum_{i=1}^{L}w_{i}^{\left(\tau \right)}b_{i}}},$$

Where $w_{i}^{\left(t\right)}\in \mathbb{C}$ represents the the $i^{\text{th}}$ element of $w^{\left(t\right)}$, and “○” represents spatial warping [20].

The three entities on the right hand side of Equation (3) are generated from three separate neural networks ${𝓖}_{\psi }:{\mathbb{R}}^{K}\to {\mathbb{R}}^{2\times N}$, ${𝓖}_{\zeta }:{\mathbb{R}}^{K}\to {\mathbb{C}}^{L}$, and ${𝓖}_{\theta }:{\mathbb{R}}^{c\times N}\to {\mathbb{C}}^{L\times N}$ such that $\phi^{\left(\tau \right)}={𝓖}_{\psi }\left(z^{\left(\tau \right)}\right)$, $w^{\left(\tau \right)}={𝓖}_{\zeta }\left(z^{\left(\tau \right)}\right)$, and $b_{1:L}={𝓖}_{\theta }(\bar{z})$, where ψ, ζ, and θ are the parameters of the three networks, and $\bar{z}\in {\mathbb{R}}^{c\times N}$ represents the static code vector of size N and with c channels. The dynamic and static code vectors, $z^{\left(1:T\right)}$ and $\bar{z}$, are optimized along with the network parameters Ξ=[ψ,ζ,θ]. We furthermore add regularization to ensure smoothness of the deformation fields $\phi^{\left(1:T\right)}$. The optimization problem solved in M-DIP then is:

(4) $${\hat{z}}^{\left(1:T\right)},\hat{\bar{z}},\hat{\psi },\hat{\zeta },\hat{\theta }=\underset{z^{\left(1:T\right)},\bar{z},\psi ,\zeta ,\theta }{\text{arg}\;\text{min}}\sum_{t=1}^{T}{\|A^{\left(t\right)}\left[\phi^{\left(t\right)}\circ \sum_{i=1}^{L}w_{i}^{\left(t\right)}b_{i}\right]-y^{\left(t\right)}\|}_{2}^{2}+\lambda_{\text{s}}{\|g_{\text{s}}\left(\phi^{\left(1:T\right)}\right)\|}_{2}^{2}+\lambda_{\text{f}}{\|g_{\text{f}}\left(\phi^{\left(1:T\right)}\right)\|}_{2}^{2},$$

with

$$\phi^{\left(t\right)}={𝓖}_{\psi }\left(z^{\left(t\right)}\right),\;w^{\left(t\right)}={𝓖}_{\zeta }\left(z^{\left(t\right)}\right),\;b_{1:L}={𝓖}_{\theta }(\bar{z}).$$

The functions $g_{s}$ and $g_{f}$ compute the finite differences along the spatial and along the frame dimension, respectively, and $\lambda_{\text{s}}$ and $\lambda_{\text{f}}$ are the corresponding regularization weights.

#### 2.2.1 Spatial dictionary generator

The spatial dictionary generator network ${𝓖}_{\theta }$ is a U-Net architecture [21]. It takes as input the static code vector $\bar{z}$ where we choose the number of channels c=2. The output of the network is L complex-valued images, represented using 2L channels, where L is an application-dependent user-defined parameter. Each convolutional block in the U-Net consists of two 2D convolutional layers, each of them followed by a leaky ReLU activation function [22] and batch normalization [23]. Downsampling is implemented using average pooling and upsampling is performed using bilinear interpolation. A detailed network architecture is described in Figure 7b.

#### 2.2.2. Temporal weights generator

The temporal weights generator network ${𝓖}_{\zeta }$ is a multi-layer perceptron (MLP) with seven fully-connected layers and leaky ReLU activation functions. The input size is K=4, and the output size is 2L. For every code vector $z^{\left(t\right)}$ the MLP generates L complex-valued weights $w^{\left(t\right)}$. A detailed network architecture is given in Figure 7c.

#### 2.2.3 Deformation field generator

The input to the deformation field generator network ${𝓖}_{\psi }$ is the same as to the temporal weights generator. First, an MLP with five fully-connected layers increases the size of the latent space from K to $N/2^{4}$. The feature vector is then reshaped to a 2D array and passed through a convolutional neural network consisting of five blocks and four upsampling operations between the blocks. Each block consists of three convolutional layers, where each layer is followed by a leaky ReLU activation function and batch normalization. Upsampling is performed using nearest-neighbor interpolation. A final convolutional layer with two output channels generates the deformation fields in x and y directions. A detailed network architecture is provided in Figure 7d.

#### 2.2.4 Implementation details

We optimize the three networks jointly for $N_{\text{iter}}=8,000$ iterations. A cosine annealing learning rate schedule [24] was used to decrease the learning rate to 0.1% of the initial learning rate over the duration of the training. We furthermore used different initial learning rates for the static components (i.e., spatial dictionary generator network ${𝓖}_{\theta }$ and static code vector $\bar{z}$) and the dynamic components of our model (i.e., temporal weights generator ${𝓖}_{\zeta }$, deformation field generator ${𝓖}_{\psi }$, and dynamic code vectors $z^{\left(1:T\right)}$). We denote these initial learning rates as $\eta_{\text{s}}$ for the static components and $\eta_{\text{f}}$ for the dynamic components.

Following Ulyanov et al. [25], we initialize the static code vector using independent and identically distributed (i.i.d.) samples from a uniform distribution, 𝒰(0,0.1), for each element ${\bar{z}}_{ij}$. We further apply noise regularization on $\bar{z}$ by adding i.i.d. Gaussian noise, $𝓝\left(0,\sigma_{n}^{2}\right)$, at each training iteration n, where $\sigma_{n}^{2}$ denotes the noise variance. This encourages solutions invariant to small perturbations. The magnitude of the added noise decreases over the training iterations, following $\sigma_{n}=\sigma_{0}\left(1-0.9n/N_{\text{iter}}\right)$, where $\sigma_{0}$ is the initial noise level. In contrast, the dynamic code vectors $z^{\left(1:T\right)}$ are initialized to a constant value of all zeros. To reduce memory usage, we process a randomly selected mini-batch of 96 consecutive frames in each training iteration. Before network optimization, the data are compressed to 12 virtual coils, and coil sensitivity maps are estimated from time-averaged k-space data using ESPIRiT [26]. All reconstructions were performed on an NVIDIA A100 GPU with 80 GB of memory.

### 2.3 Baseline methods

We compare our method to LR-DIP [18], which is a state-of-the-art DIP-based method for reconstructing cardiac cine MRI. An implementation for LR-DIP was provided by the original authors and adjusted for Cartesian data. We use 5% dropout rate, which was determined by the authors to provide the best image quality at 1.5 T. The rank $k_{\text{lr}}$ is chosen depending on the application and such that it is considerably lower than the number of image frames. The default depth of the spatial and temporal U-Net, i.e., the number of downsampling and upsampling operations, in LR-DIP is five. We use this value in our experiments unless the dimensionality of the data requires a lower value.

We further compare to reconstructions obtained using the low-rank plus sparse (L+S) method [27]. Reconstructions of in-vivo cine data are furthermore compared to a supervised deep learning reconstruction for cardiac cine MRI, called CineVN [28].

### 2.4 Experiments and data

#### 2.4.1 Phantoms

Seven datasets from real-time cardiac cine MRXCAT phantoms with different anatomies were simulated [19] with an isotropic resolution of 2 mm and slice thickness of 8 mm. Each simulated cine was 9 s long and contained two breathing cycles with 4.5 s each and 10 cardiac cycles of random durations between 0.8 s and 1.0 s. At a temporal resolution of 30 ms, the simulated cine series consisted of 300 image frames each. Complex multi-coil k-space data for 12 coils was simulated and random complex-valued Gaussian noise was added to achieve a signal-to-noise ratio of 10dB on the individual coil images. The simulated k-space data was then retrospectively undersampled using a variable density golden ratio offset (GRO) Cartesian sampling pattern [29] with an acceleration factor of eight.

Reconstructions were performed using M-DIP, LR-DIP, and L+S and were compared to the noise-free ground-truth simulation in terms of peak signal-to-noise ratio (PSNR), normalized root mean square error (NRMSE), and structural similarity index (SSIM). The hyperparameters for M-DIP and LR-DIP are given in Table 1.

#### 2.4.2 Real-time cine

Free-breathing real-time cine data were collected in 27 unique clinical patients on a 1.5 T scanner (MAGNETOM Sola, Siemens Healthineers, Forchheim, Germany) using a balanced steady-state free precession (bSSFP) sequence. The acquisitions were prospectively undersampled using a golden ratio offset (GRO) sampling pattern [29] with an acceleration rate between seven and ten. Between 196 and 212 phases were acquired in each cine with a temporal resolution between 47 ms and 51 ms. In total, each cine was 10.0 s long, which usually covered two to four breathing cycles and 10 to 15 cardiac cycles. In-plane resolution varied between 2.04 mm and 2.84 mm, slice thickness between 6 mm and 8 mm, and a flip angle of 70° was used in all protocols. Twenty-six slices were acquired in the short-axis view and one in the long-axis view.

Reconstructions were obtained using M-DIP, LR-DIP, L+S, and CineVN. The hyperparameters for M-DIP and LR-DIP are given in Table 1. Two readers with more than ten years of experience in CMR scored all reconstructions in terms of “image sharpness” and “perceived noise and artifacts” on five-point Likert scales (1=Nondiagnostic, 2=Poor, 3=Fair, 4=Good, 5=Excellent).

#### 2.4.3 Single-shot LGE

Thirty-three free-breathing single-shot LGE image series were collected from 20 unique clinical patients on the same 1.5 T scanner as the cine data. For acquisition, a phase-sensitive inversion recovery (PSIR) sequence [30] with inversion times between 270 ms and 410 ms and a flip angle of 40° was used. In-plane resolution varied between 1.37 mm and 1.88 mm, with a slice thickness set at 8 mm. A GRO sampling pattern was used for prospective undersampling with an acceleration rate between four and six. Each series consisted of 32 T1-weighted image frames, each representing a single-shot acquisition that was prospectively triggered using a proprietary ECG signal. Fifteen slices were acquired in the short-axis view and 18 in the long-axis view.

Reconstructions were obtained using M-DIP, LR-DIP, and L+S. The hyperparameters for M-DIP and LR-DIP are given in Table 1. Note that we use a lower rank (L in M-DIP, $k_{\text{lr}}$ in LR-DIP) compared to the cine data due to the smaller number of image frames in the LGE series. We also reduce the depth of the temporal basis network in LR-DIP for this reason. Furthermore, we do not use regularization along the temporal dimension of the deformation fields in M-DIP $\left(\lambda_{\text{f}}\right)$ because consecutive frames are not expected to be similar in their breathing motion state. Reconstructions were scored in terms of “clarity of pertinent myocardial features” on a five-point Likert scale (1=Nondiagnostic, 2=Poor, 3=Fair, 4=Good, 5=Excellent).

## 3 Results

### 3.1 Phantom study

**Figure 2:**
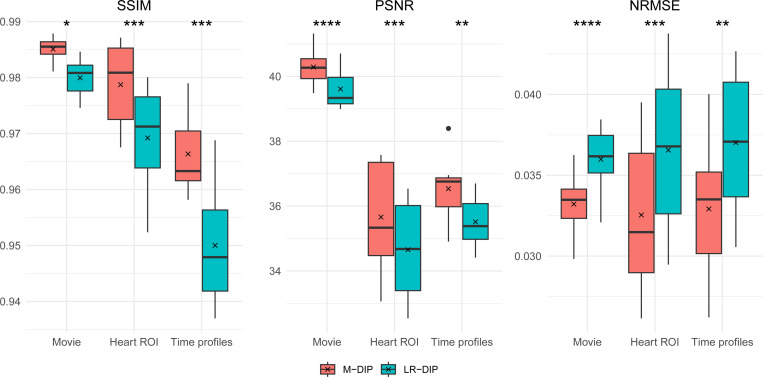
M-DIP and LR-DIP results of the phantom study with results of Student’s t-tests. * indicates *p* ≤ 0.05, ** indicates *p* ≤ 0.01, * * * indicates *p* ≤ 0.001, and * * ** indicates *p* ≤ 0.0001.

**Figure 3:**
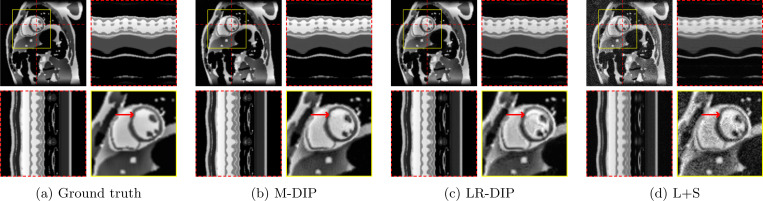
Exemplary MRXCAT phantom reconstructions. Each sub-figure illustrates an end-diastolic frame, temporal profiles, and a close-up on the heart. Red arrows show an area with an artifact in LR-DIP that is suppressed in M-DIP.

The results of the phantom study are given in Table 2. SSIM, PSNR, and NRMSE were computed on the full cine movies, on a region of interest (ROI) around the heart, and on temporal profiles through the center of the heart. M-DIP achieved the best scores in all metrics. The results of M-DIP and LR-DIP are additionally illustrated in Figure 2, and Student’s t-tests were performed to evaluate them for statistical significance. All indicated p-values are significant (α=0.05) after correction for multiple comparisons using the Holm-Bonferroni method. Reconstruction time was approximately 40 min for M-DIP and 70 min for LR-DIP. Exemplary reconstructions of one phantom are illustrated in Figure 3.

### 3.2 In-vivo study

**Figure 4:**
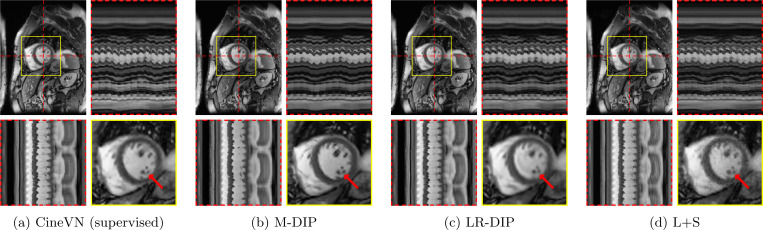
Exemplary real-time cine reconstructions. Each sub-figure illustrates an end-diastolic frame, temporal profiles, and a close-up on the heart. Red arrows show an area where differences in image sharpness are particularly apparent.

**Figure 5:**
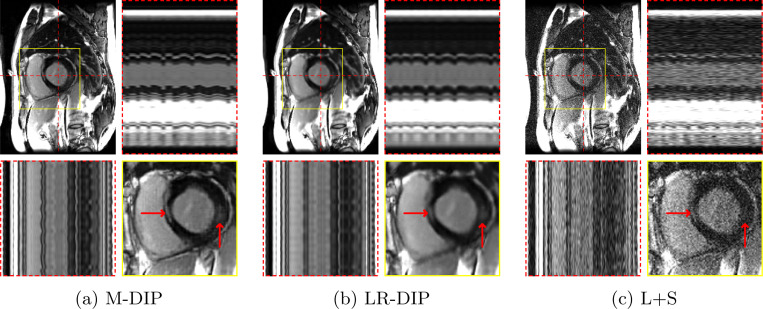
Exemplary free-breathing single-shot LGE reconstructions. Each sub-figure illustrates one frame, temporal profiles, and a close-up of the heart. Red arrows show details that are better visible in M-DIP compared to LR-DIP.

**Figure 6:**
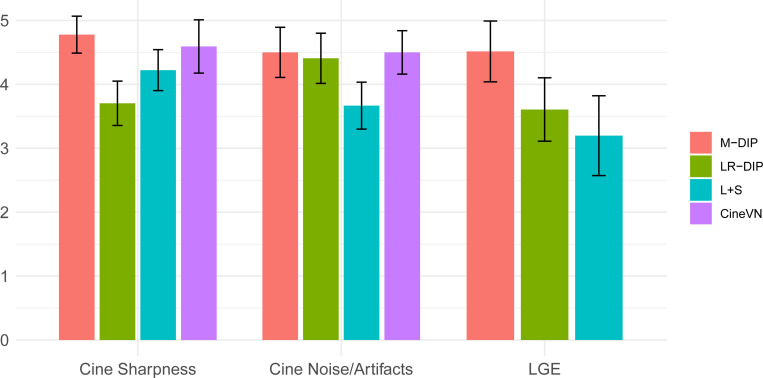
Boxplots with the scoring results for the in-vivo studies

**Figure 7:**
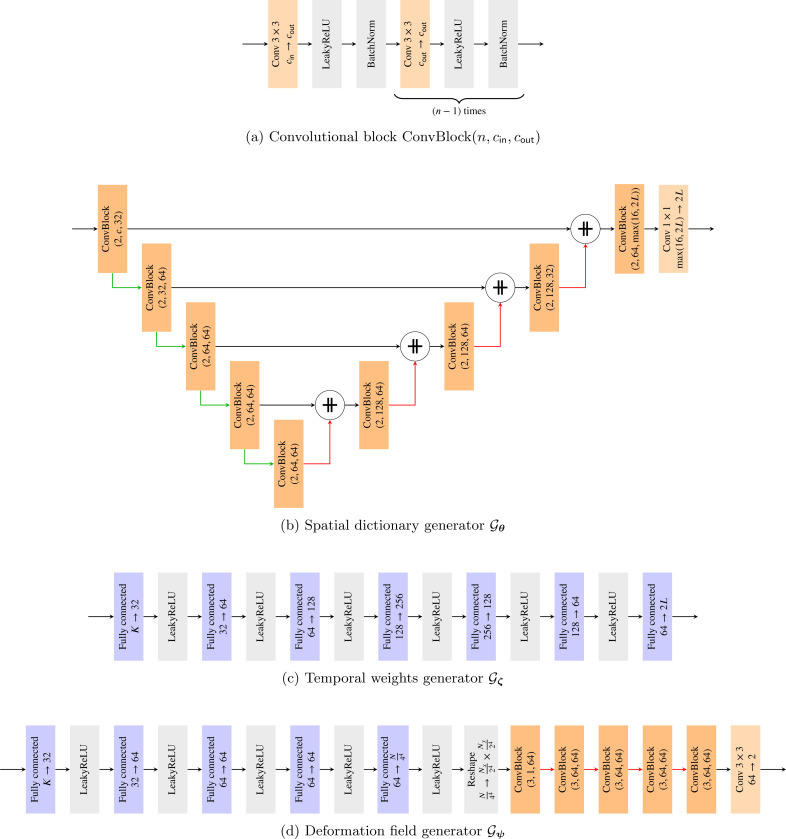
Detailed architecture of the three generator networks. n, $c_{\text{in}}$, and $c_{\text{out}}$ are the number of convolutional layers, input channels, and output channels, respectively, in the convolutional blocks. $N_{x}$ and $N_{y}$ are the number of image pixels along the spatial dimensions. Green arrows denote average pooling with kernel size 2 × 2 and red arrows denote interpolation by a factor of two. ⧺ denotes concatenation along the channel dimension.

Figure 4 and Figure 5 illustrate the results for an exemplary real-time cine dataset and a free-breathing single-shot LGE dataset, respectively. Scoring results are given in Table 3 and Figure 6. M-DIP achieved highest scores in all considered criteria. Sharpness of real-time cines was scored significantly higher in M-DIP than in any other method. M-DIP and CineVN were scored equally in terms of noise/artifacts in the real-time cines, where only L+S received significantly lower scores than the other methods. In free-breathing single-shot LGE, M-DIP was scored significantly higher than both LR-DIP and L+S. Cine reconstruction took approximately 30 min with M-DIP and 45 min with LR-DIP, while LGE reconstruction took approximately 15 min with M-DIP and 10 min with LR-DIP.

## 4 Discussion

In this work, we propose a DIP-inspired method called M-DIP and evaluate it using simulated and patient data for real-time cine, as well as patient data for single-shot LGE imaging. M-DIP is an instance-specific approach that does not require training data. Unlike other DIP methods, M-DIP can model both motion and content variations, making it suitable for a wide range of dynamic applications.

In the first study, we simulated multi-coil undersampled k-space data using free-breathing real-time cardiac cine MRXCAT phantoms. M-DIP outperforms both L+S and the recently proposed LR-DIP method in terms of PSNR, NRMSE, and SSIM. The performance gap between M-DIP and L+S is substantial, as L+S produces images with high levels of noise. While the performance advantage of M-DIP over LR-DIP is more moderate, visual inspection reveals that LR-DIP consistently introduces motion blurring around the myocardium. In contrast, M-DIP captures cardiac motion more accurately, as shown in Figure 3.

In the second study, we reconstructed prospectively undersampled free-breathing real-time cine data in clinical patients and reconstructed them using M-DIP, LR-DIP, L+S, and a recently proposed supervised learning method (CineVN). As no ground truth is available, a reader study was performed by two experienced readers.

In terms of noise and artifacts, M-DIP and CineVN performed the best, while the scores for LR-DIP were insignificantly lower. L+S received the lowest scores, exhibiting high levels of noise in the reconstructions. In terms of image sharpness, M-DIP reconstructions received significantly higher scores, while LR-DIP received the lowest scores. Visual inspection again reveals blurring in the LR-DIP reconstructions mainly around the myocardium, suggesting a limitation of the low-rank model in the presence of complex motion over multiple respiratory and cardiac cycles in a large field of view, as seen in Figure 4. The M-DIP framework in contrast allows generating a high-resolution template image for each frame which is then deformed to the respective motion state. The high scores for noise and artifacts in M-DIP and LR-DIP furthermore highlight the implicit capability of DIP-based methods to generate natural-looking images [13].

Notably, M-DIP significantly outperformed CineVN in terms of image sharpness, even though it did not benefit from training data. Since acquiring fully-sampled training data for real-time cine is infeasible, CineVN was exclusively trained on breath-held segmented acquisitions, which may have limited its performance on free-breathing real-time data. In contrast, M-DIP is unsupervised and directly learns the physiological motion and content variations from the undersampled data itself.

In a further in-vivo study, we applied M-DIP to free-breathing single-shot LGE data, thus demonstrating our method’s suitability for dynamic applications beyond cine imaging. The clarity of myocardial features was rated highest in the M-DIP reconstructions, with a substantial performance gap over LR-DIP and L+S. Similar to real-time cine, LR-DIP reconstructions are blurred, while L+S reconstructions exhibit high levels of noise.

One of the limitations of this work includes longer reconstruction times, taking several tens of minutes for one image series on a single GPU. Also, we note that optimizing M-DIP with its three subnetworks requires a careful selection of hyperparameters. Future efforts will focus on reducing the computational time of M-DIP and extending it to other CMR applications, including perfusion and mapping.

## 5 Conclusions

We have proposed, implemented, and evaluated an unsupervised method, M-DIP, for dynamic image reconstruction from highly undersampled data. Results from simulated cine data indicate that M-DIP surpasses competing methods in image quality. Image quality assessments for real-time cine and LGE reveal that M-DIP outperforms other unsupervised methods and performs comparably to a recent state-of-the-art supervised learning approach.

## Figures and Tables

**Table 1:** Reconstruction hyperparameters. M-DIP parameters: Number of dictionary elements (L), regularization weights for spatial and temporal smoothness regularization on the deformation fields ($\lambda_{\text{s}}$ and $\lambda_{\text{f}}$, respectively), initial noise regularization scale $\left(\sigma_{0}\right)$, and initial learning rates for static and dynamic model components ($\eta_{\text{s}}$ and $\eta_{\text{f}}$, respectively). LR-DIP parameters: Rank of the low-rank system ($k_{\text{lr}}$) and depth of the spatial and temporal basis U-Nets ($d_{\text{S}}$ and $d_{\text{T}}$, respectively).

	M-DIP						LR-DIP		
Dataset	L	$\lambda_{\text{s}}$	$\lambda_{\text{f}}$	$\sigma_{0}$	$\eta_{\text{s}}$	$\eta_{\text{f}}$	$k_{\text{lr}}$	$d_{\text{S}}$	$d_{\text{T}}$
Phantom	16	0.02	0.02	0.02	1·10^−3^	1·10^−3^	64	5	5
Cine	16	0.1	0.05	0.05	1·10^−3^	1·10^−3^	64	5	5
LGE	8	0.1	0	0.1	5·10^−4^	1·10^−3^	12	5	4

**Table 2:** Results of the phantom study. The best value in each column is written in boldface.

	Movie			Heart ROI			Time profiles		
	SSIM	PSNR	NRMSE	SSIM	PSNR	NRMSE	SSIM	PSNR	NRMSE
M-DIP	**0.985**	**40.3 dB**	**0.0332**	**0.979**	**35.7 dB**	**0.0325**	**0.966**	**36.5 dB**	**0.0329**
LR-DIP	0.980	39.6 dB	0.0360	0.969	34.7 dB	0.0366	0.950	35.5 dB	0.0370
L+S	0.701	30.2 dB	0.1080	0.826	26.2 dB	0.1020	0.658	26.9 dB	0.1050

**Table 3:** Mean scoring results for the in-vivo studies.

	Real-time cine		Single-shot LGE
	Sharpness	Noise/Artifacts	Clarity of myocardial features
M-DIP	**4.78**	**4.50**	**4.52**
LR-DIP	3.70 ***	4.41^(ns)^	3.61 ***
L+S	4.22 ***	3.67 ***	3.20 ***
CineVN	4.59 *	**4.50** ^(ns)^	—

p-values of Student’s t-tests with respect to M-DIP are given, where (ns) indicates *p* > 0.05,

* indicates *p* ≤ 0.05,

** indicates *p* ≤ 0.01, and

*** indicates *p* ≤ 0.001. The best value in each column is written in boldface.
